# Effectiveness and cost-effectiveness of a single home-based fall prevention program: a prospective observational study based on questionnaires and claims data

**DOI:** 10.1186/s12877-024-05586-x

**Published:** 2024-12-28

**Authors:** Karin Niedermann, André Meichtry, Barbara Zindel, Markus J. Ernst, Valérie Krafft, Renato Mattli, Irina Nast, Simon Wieser, Markus Wirz, Beatrice Brunner

**Affiliations:** 1 ZHAW Zurich University of Applied Sciences, School of Health Sciences, Katharina-Sulzer-Platz 9, Winterthur, 8401 Switzerland; 2 BFH Bern University of Applied Sciences, School of Health Professions, Bern, Switzerland; 3 Swiss League Against Rheumatism, Zurich, Switzerland; 4 ZHAW Zurich University of Applied Sciences, Winterthur Institute of Health Economics, Winterthur, Switzerland

**Keywords:** Falls, Prevention, Effectiveness, Cost, Cost-effectiveness

## Abstract

**Background:**

Fall prevention programmes are essential interventions in societies with aging populations. This study assessed the fall rate and other health outcomes, as well as the cost-effectiveness of a home-based fall prevention programme for community-dwelling older people. In a single home visit, trained physical or occupational therapists performed fall risk assessments, eliminated environmental risk factors, and provided tailored exercises.

**Methods:**

A prospective, longitudinal observational study was performed with participants of a fall prevention programme who agreed to be followed-up over one year. Baseline data included self-reported falls one month and one year before the intervention. Participants were monitored through bi-monthly telephone calls, assessing their number of falls, fear of falling (using the Falls Efficacy Scale-International (FES-I), quality of life (using the EuroQuol-5 Dimensions-5 Levels, EQ-5D-5L), and physical activity (in minutes per week). Statistical analysis of the data used a Generalized Estimating Equations (GEE) Poisson-Modell for number of falls and a Linear Mixed Model (LMM) for fear of falling, quality of life and physical activity. In addition, health insurance claims data were used to compare the number of medically treated falls in the year before and after the intervention, as well as the related healthcare spending. Cost-effectiveness of the programme versus usual care was estimated as cost per prevented medically treated fall.

**Results:**

Overall, 639 person-years of observation time were analyzed. Participants had a mean age of 81.8 years (+/- 5.2) and 59% were female. On average, the fall rate decreased from 1.35 to 1.02 per person-year, or -23.9% (95%CI from -35.92 to -9.67), fear of falling decreased by -1.27 points (95%CI from −1.50 to -1.05), quality of life improved by -0.88 points (95%CI from −1.09 to -0.68), and physical activity increased by 9.87 min per day (95%CI from 5.65 to 14.09). Analysis of claims data showed a 48.0% reduction (95%CI from 30.5% to 61.0%) in medically treated falls. The average cost per prevented medically treated fall was estimated at approximately 1,353 USD, with a 50% probability of the intervention being cost saving.

**Conclusions:**

This fall prevention programme with a single home visit was found to be effective and cost effective. Health policies should establish such a model as a reimbursed standard care to assist in combatting the increasing burden of falls on individuals and societies.

**Supplementary Information:**

The online version contains supplementary material available at 10.1186/s12877-024-05586-x.

## Background

Falls in older adults are a major healthcare problem in high income countries [1, 2]. About 30% of adults aged 65 years and older experience at least one fall per year [3, 4] and the risk of falls increases substantially with age [1, 2, 5]. In the US, about 10% of adults aged 65 years and older report at least one fall related injury per year [3]. Falls are responsible for 5.7% of years lived with disability in people aged 70 years and above in the United States (US), and an even greater share in Western Europe,  of 7.5% [5].

Switzerland is ranked among the top three European countries, with a fall incidence of 23–33% [2, 4], a fall-related death rate of 3.3% [2], and a share of 11.0% in years lived with disability in adults aged 70 and above [2, 5]. Typical fall injuries include hip fractures, brain injuries, and upper limb injuries, often requiring surgery, long hospital stays and rehabilitation. Falls often lead to loss of independence [1, 5]. First and recurrent falls also increase fear of falling and physical inactivity, leading to a vicious cycle [6]. Falls pose severe threats to the quality of life and autonomy.

The severe consequences of individual falls multiply to substantial challenges for healthcare systems and high socio-economic costs. US medical costs of falls in adults over 65 years have been estimated at $50 billion [7]. For Switzerland, the total economic cost of falls in older adults was estimated at 6.6 billion Swiss Francs (CHF) [8].

Falls are usually caused by a combination of intrinsic and extrinsic risk factors [9], many of which are modifiable through targeted interventions. Fall prevention programmes (FPPs) have potential to substantially reduce the burden of disease on individuals and societies. According to the World Guidelines for Falls Prevention and Management for Older Adults, all older people should be advised on falls prevention and physical activity; and falls risk assessments are also recommended, with those identified as ‘at high-risk’ should get personalized interventions [10]. FPPs should be multidisciplinary and multifactorial, including exercises, medication adaptation, and environmental change [11]. Exercise programmes to reduce falls should primarily involve balance and functional exercises [12]. A recent systematic review of economic evaluations of FPPs showed that home safety assessments and modifications were the most cost-effective type of programme for older adults [13].

The Swiss League Against Rheumatism (SLAR) developed the multidimensional, home-based FPP ‘Safe through Daily Life’ (SDL), based on the Australian ‘Stay On Your Feet’ FPP [14–16] which basically already applied the above-mentioned evidence and recommendations: Trained physical or occupational therapists visited community dwellers at their home and performed a fall risk assessments, eliminated environmental risk factors, and provided tailored exercises within one single visit (more details see ‘methods/participants’). The SDL was piloted in 2013 [17] and subsequently implemented nationwide. The SDL was not covered by Swiss mandatory health insurance (MHI) as current regulations exclude coverage by MHI for most prevention and health promotion services. However, three large health insurance companies, insuring 2’638’800 persons (31% of the total population in Switzerland), agreed to cover the SDL participation fee for older insurees with an additional non-mandatory health insurance.

The aims of this study were to evaluate: (1) The effectiveness of SDL in reducing the number of falls; and (2) Its cost-effectiveness versus usual care, in terms of cost per prevented severe fall in terms of requiring medical treatment, based on claims data. In case of successful falls reduction and cost-effectiveness, this may be relevant for health insurers to establish sustained coverage of the SDL costs. Data sources include both survey data and medical claims data.

## Methods

### Design

**Fig. 1:**
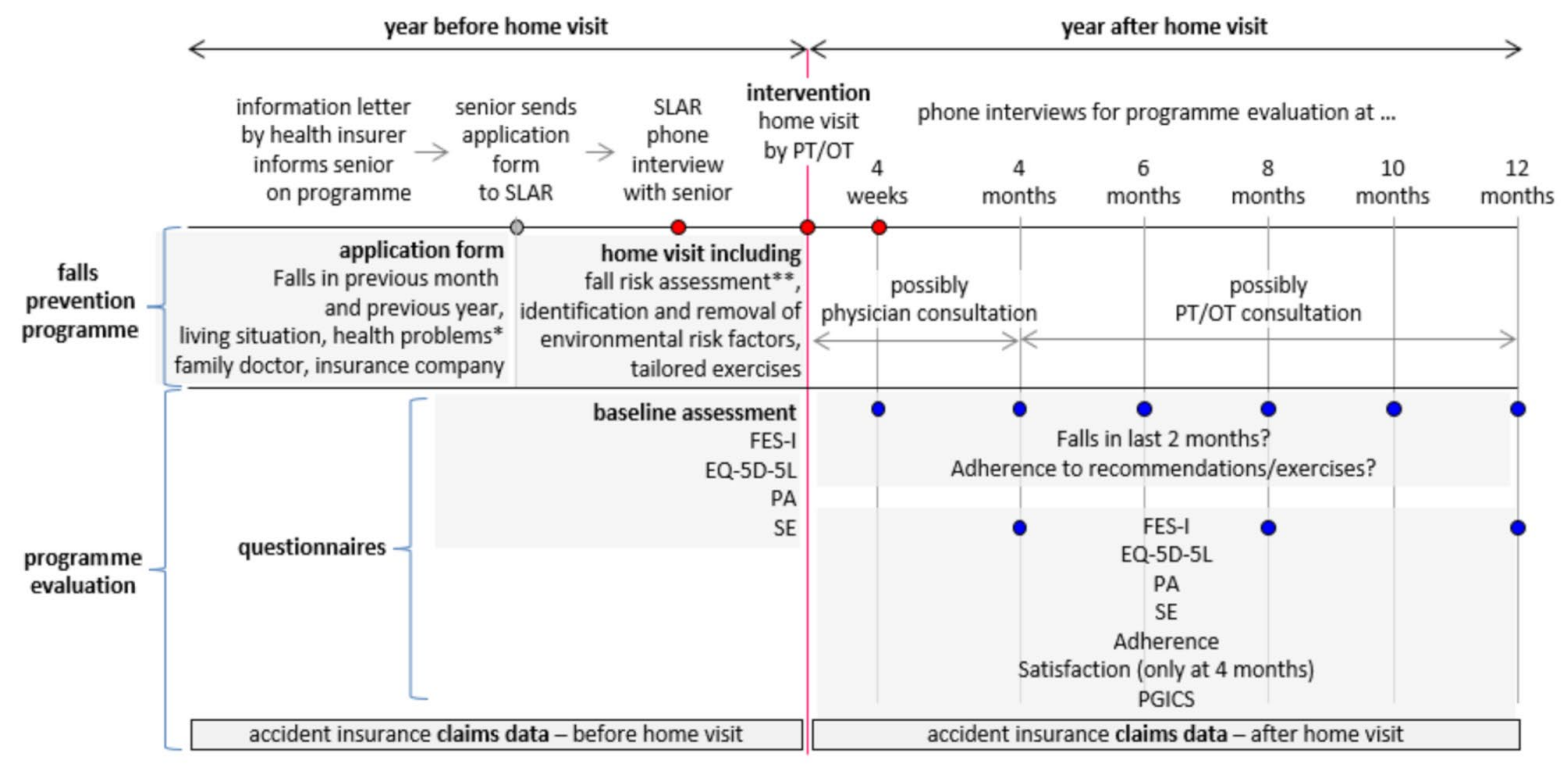
Overview of study procedures and assessments SLAR: Swiss League Against Rheumatism; PT: physiotherapist, OT: occupational therapist, FES-I = Falls Efficacy Scale-International; EQ-5D- 5 L = EuroQuol-5 Dimensions-5 Levels; PA = Physical activity, minutes/week spent ‘walking’; SE = Self-efficacy, related to the given recommendations and the instructed exercises; PGICS = Patient Global Impression of Change Scale *health problems = need of visual aid, balance problems, pain while walking, problems getting up from chair, dizziness, use of walking aid **fall risk assessment: (a) checklist on health behaviors and intrinsic risk factors for falls; (b) three functional tests: 1) FCST = (Five) Chair Stand Test; TUG = Timed Up and Go (including TUGmot = TUG with additional motor task; and TUGcog = TUG with additional cognitive task); 3) Getting-up-from-floor

A prospective, longitudinal observational study was performed between 2017 and 2020. Participants were monitored for one year after the SDL home visit. Additionally, medically treated falls and the costs were identified in the health insurance claims data in the years before and after the home visit. Figure 1 gives an overview of the intervention and the evaluation.

### Participants

The three supporting health insurance companies sent regular letters to all their clients, who fulfilled the inclusion criteria (see below), inviting them to participate in the SDL programme. Thus, persons interested in the SDL had to contact the SLAR themselves by mail or telephone. The SLAR assessed the in- / exclusion criteria to the FPP and the number of falls in the previous one month and one year in a short interview. Inclusion criteria were community dwellers, aged 70 years or older, disposing of an additional non-mandatory health insurance by the same health insurer for at least one year; exclusion criteria were cognitive limitations and planned move to a nursing home. Additionally, all persons admitted to the SDL were invited to the study, and included, provided they gave written informed consent to five bi-monthly assessments by telephone and use of their health insurance data for cost analysis, either by post-mail or at the beginning of the home visit.

### SDL home visit / initial assessment

In the single home visit of 60–90 min by a physical or occupational therapist, a detailed assessment of each participant’s risk of falling was performed, using (a) A checklist of health behaviors, e.g. smoking and intrinsic fall risk factors; (b) Three functional tests: Five Chair Stand Test (FCST); Timed Up and Go (TUG); Get-up-from-floor; (c) Falls Efficacy Scale-International (FES-I) short version [18]; and (d) Tour through the home to identify extrinsic fall risk factors. The subsequent intervention involved eliminating the extrinsic fall risk factors, e.g., fixing carpets, and instruction of maximal five individually tailored exercises. After 4 weeks, the physio-/occupational therapist called the participant to discuss any further needs.

### Study outcomes and assessments

The primary outcome was fall rate, assessed by the self-reported number of falls and number of medically recorded severe falls during the 1-year observation period after the home visit.

Secondary outcomes were fear of falling, quality of life, physical activity, adherence and global impression of change. Thus, fear of falling, using the FES-I, already established in the SDL, was complemented by (a) Quality of life using the EuroQuol-5 Dimensions-5 Levels, EQ-5D-5L questionnaire, including EQ-5D-VAS (rating health on a 0-100 VAS) [19]; (b) Physical activity (PA), minutes per week spent ‘walking’, derived from the International Physical Activity Questionnaire IPAQ: [20]; (c) Adherence to the recommendations and exercises (from 0 = never to 5 = always; d) Global impression of change (0 = much worse, 5 = no change, 10 = much better) using the Patients’ Global Impression of Change Scale (PGICS) [21]. Baseline data for c) and (d) were assessed at 4 months: Self-efficacy for performing the recommendations and the exercises (from 0 = no confidence to 10 = highest confidence) [22] was assessed at the home visit, satisfaction with the SDL (very satisfied – satisfied – not satisfied) was assessed at 4 months.

The assessments were effectuated through bi-monthly telephone calls by SLAR staff: at months 4, 8 and 12, participants reported on all endpoints, with calls lasting 20–40 min; at months 1, 6 and 10, only falls and adherence data were collected, with calls lasting 5–15 min. Participants were also requested to document falls immediately in a simple daily calendar. See Fig. 1 for overview.

### Medical cost of falls

The Swiss healthcare system is based on transparent, uniform nationwide prices for mandatory health and accident insurance. This simplifies economic health evaluations compared to countries with more non-transparent prices, such as the US.

Medical treatment costs were drawn from claims data of three large insurance companies participating in the study. These companies provided mandatory health insurance and mandatory accident insurance to the study participants. Accident insurance covers injuries caused by accidents, thus allowing us to study the healthcare costs related to accidents separately from disease-related healthcare costs. Cost included all inpatient and outpatient health services and medications covered by accident insurance. The study covered the years 2017 to 2020, with prices held constant throughout this period. Falls requiring medical treatment were identified from accident notifications compiled by the treating physicians describing the circumstances of the accident. The costs of severe falls were limited to the two-month period after the fall, to avoid possible contamination by treatment costs of recurrent falls.

### Program costs

The cost per participant were CHF 500 (about $500) [23].

### Statistical analysis

Effectiveness: The change in the fall rate (number of falls / person-year) was calculated by comparing the number of falls in the year before and after the home visit. The mean number of falls before the visit was estimated, based on self-reported falls in (a) the year, and (b) the month (multiplied by 12) before the visit. It is unknown to what extent recall bias can result in the underreporting of retrospectively self-reported falls compared to prospectively calendar-reported falls. Several studies on community dwellers aged over 70 years described underreporting of falls after various periods of time to different extents, i.e., 25% underreporting of falls in a 3-month recall [24], 44% over a six-month period [25], 23% underreporting in a 12-months recall [26], and 13% over a 12-month period [27]. Computing a weighted mean for a 12-month recall rate estimate from these reported rates, using the observation times as weights, results in a 12-month recall rate estimate of 0.77. Using only the reported 12-month recall rates from Sanders et al. [26] and Cummings et al. [27] would result in a recall rate estimate of 0.82. Assuming unbiased 1-month reporting, a mean of the adjusted 12-month reporting and the 1-month (times 12) reporting would represent an estimate of the number of falls in the year before the intervention. A 2:1 weighting of the two self-reportings was used, thus contributing more weight to the smaller 1-year reporting relative to the larger 1-month (times 12) reporting. This results in less falls in the year before the intervention, thus generating a conservative estimate of the fall rate reduction after the intervention.

A Poisson generalized estimating equation (GEE-Poisson) was fitted to the data for number of falls. Possible confounding by, and interactions with, age, sex, living situation and health problems, were assessed with comparisons of the respective models. Linear Mixed Models (LMM) were fitted for the secondary outcomes of fear of falling, quality of life, PA and global impression of change. In the models fitted to the secondary outcomes, we accounted for individual variability by specifying random intercepts for each ID. The fixed effects included the timepoint of data collection, age, use of walking aids, ability to rise from a chair, dizziness, and pain while walking.

All analyses were performed using the R statistical software R version 4.0.3 (2020-10-10) [28].

Cost effectiveness: In the cost-effectiveness analysis the cost per prevented severe fall, defined as a fall requiring medical care, was assessed from an accident insurance perspective. The analysis was based on a comparison of the number of severe falls and related medical costs in the year before and after the home visit. No discounting was needed since the time horizon was limited to the study year.

Since the probability of fall rises with increasing age, the number of falls in the year after the intervention was adjusted for the ageing of the participants over one year to give an estimated number of falls. This effect was estimated based on the yearly number or falls reported in the Swiss Health Survey (SHS) 2017, a large population survey covering over 22,000 non-institutionalized people. The model’s fit was assessed using adjusted R-squared, F-value, and *p*-value metrics. A simple linear model provided the best fit.

Cost effectiveness was assessed by the incremental cost effectiveness ratio (ICER), representing the monetary cost of the falls prevented by the program. It is calculated by dividing the net cost of insurers by the number of prevented severe falls. Model comparison showed that the number of prevented severe falls was not confounded by age, sex, living situation, or health problems. The net cost was calculated as the difference between total program cost and prevented health care costs (number of prevented falls × average treatment cost of falls). The Wilcoxon Rank-Sum Test was used to compare costs. The robustness of the estimated results was assessed based on the estimated confidence intervals.

## Results

**Fig. 2:**
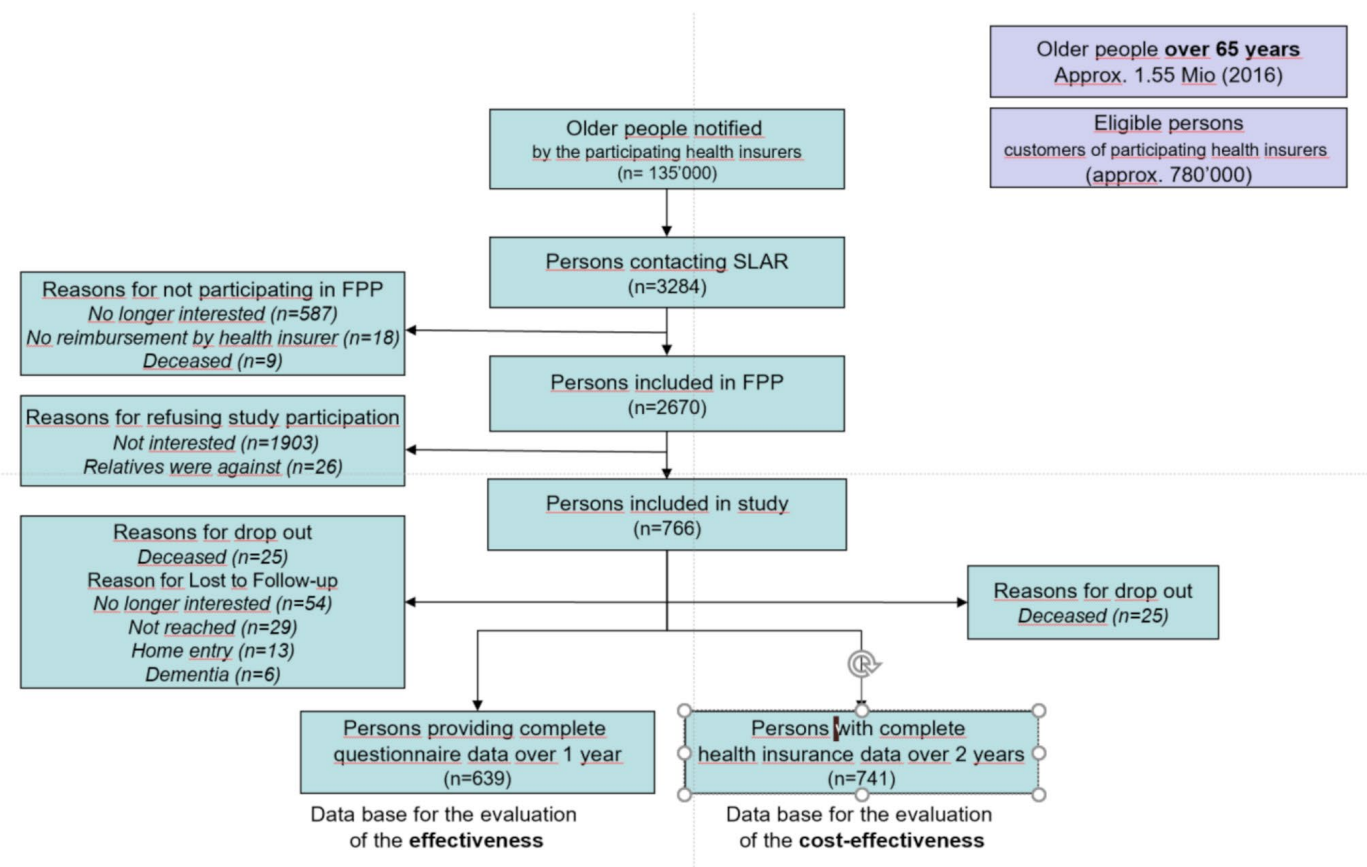
Study flow SLAR = Swiss League Against Rheumatism

Of the 2,670 older people included in the SDL during the study period, 766 (28.7%) participated in the study. Complete survey data was available for 639 (83.4%) participants for the analysis of effectiveness. For the cost-effectiveness analysis, accident insurance claims for 741 (96.7%) participants were acquired (Fig. 2).

The latter number is higher because there was no loss due to follow-up in the claims data, except for 25 participants who died during the follow-up period. See Table 1 for demographic and clinical characteristics.

**Table 1:** Demographic and clinical characteristics of study participants

Characteristics	Study participants with 1- year follow-up (*n* = 639) Effectiveness sample, Survey data	Study participants (*n* = 741) Cost-effectiveness sample, Health insurance data
Females, n (proportion)	376 (59%)	454 (61%)
Age in years, mean, SD (range)	82,5,18 (57–97)	80,5 (57–97)
Living situation - alone	335 (52%)	Not observed
Health problems		Not observed
Use of visual aids Use of walking aids Pain when walking Dizziness/vertigo Problems getting up from chair Problems with balance	554 (87%) 71 (11%) 325 (51%) 206 (32%) 208 (33%) 344 (54%)	
Five Chair Stand Test (FCST) -		Not observed
ability to perform, yes / no	442 / 197	
Timed Up and Go (TUG)		Not observed
Ability to perform in below 15 s (Yes / No / NA)		
(TUG)	531 / 106 / 2	
TUG + additional motor task	533 / 93 / 13	
TUG + additional cognitive task	360 / 270 / 9	
Getting-up-from-floor (Yes / No / NA)	448 / 75 / 116	Not observed

SD = Standard Deviation; NA = Not Applicable

### Effectiveness

A total of 652 falls occurred during the year following the home visit. Compared to the 855 estimated reported falls before the home visit this reflects a reduction of 203 falls, corresponding to an absolute fall rate reduction of -0.33 and a relative fall rate reduction of 23.9% (Table 2). A sensitivity analysis, using the mean of the 12-month recall rate estimates based on Sander 2015 and Cummings 1988 for adjusting the 12-month record, resulted in a less conservative relative fall rate reduction of 38% [26, 27].

Model comparison showed that fall rate reduction was neither modified nor confounded by age, sex, living situation and health problems. Improvements in fear of falling, health-related quality of life, and physical activity were small, but significant (0.05 significance level, corresponding to 95% confidence level), at all follow ups. The perceived health related quality of life improved at 4 and 8 months, but not at 12 months. At 12 months, PGICS remained unchanged, and adherence decreased slightly. Satisfaction with the SDL at 4 months after the home visit was high with 99% of participants being satisfied or very satisfied (Table 2).

**Table 2:** Changes in primary and secondary endpoints

**Questionnaire data (for effectiveness estimation)**			
**Endpoint**	**Baseline at home visit (SD)**	**One year after home visit (SD)**	**Change (95% CI)**
Total number of falls (12 months recall) / Fall rate (= number of falls / person-year)	599/0.94		
Total number of falls (1 month recall, multiplied by 12) / Fall rate (= number of falls / person-year)	1260/1.98		
Total number of falls (weighted 2:1 at individual level)	855	652	-203
Fall rate (= number of falls / person-year)	1.35	1.02	Relative rate reduction − 23.92% (-35.92 to -9.67) ^d)^
Fear of falling (FES-I), Total score 0–28; 0 = best, 28 = worst)	10.36 (3.27)	9.09 (2.53)	-1.27 (-1.50 to -1.05) ^d)^
Quality of life (EQ-5D-5L), Total score 0–25 (0 = best, 25 = worst)	8.65 (2.69)	7.76 2.82	-0.88 (-1.09 to -0.68) ^d)^
Quality of life (EQ-5D-VAS), Scale 0-100 (0 = worst, 100 = best imaginable health)	74.1 (16.1)	73.8 (15.9)	-0.36 points (-1.59 to 0.87)
Physical activity (minutes/day)	42.27 (32.20)	52.14 (43.75)	9.87 minutes (5.65 to 14.09) ^d)^
Adherence ^a)^ (1 = never, 2 = seldom, 3 = sometimes, 4 = most of the time, 5 = always)	Median = 4 (IQR = 2)	Median = 3 (IQR = 3)	-0.50 (pseudo-median) (-0.99 to -0.5) ^d)^
Patient’s Global Impression of Change Scale (PGICS) Score 0–10 (5 = no change, 1–4 = worse; 6–10 = better)	5.97 (1.56) ^a)^	6.22 (1.56)	0.25 (0.09 to 0.42) ^d)^
Self-efficacy for recommendations Scale 0–10 (0 = no confidence at all; 10 = highest confidence)	7.52 (2.78)	NA	NA
Self-efficacy for exercises Scale 0–10 (0 = no confidence at all; 10 = highest confidence)	8.41 (2.53)	NA	NA
Satisfaction ^a)^			
Very satisfied		45% ^a)^	
Satisfied		54%	
Unsatisfied		1%	
**Claims data (for cost-effectiveness estimation)**			
**Endpoint**	**Before home visit**	**One year after home visit**	**Change (95% CI)**
Predicted additional falls due to aging in year after home visit		6	(3, 8)
Total number of severe falls (medically treated)	124	67	-63 Relative rate reduction including additional falls -48.46%
Number of participants with falls	111 ^b)^	55 ^c)^	-66 Relative Rate Reduction -49.55%

SD = Standard Deviation; CI = Confidence Interval; Notes: ^(a)^ assessed 4 months after the intervention. ^(b)^ Number of falls by number of participants: 1 (*n* = 100), 2 (*n* = 9), 3 (*n* = 2). ^(c)^ Number of falls by number of participants: 1 (*n* = 45), 2 (*n* = 8), 3 (*n* = 2). ^d)^significant change.

### Cost effectiveness

In the year before the home visit, 111 participants had a total of 124 severe falls (see Table 2). Based on SHS data from respondents aged 65 years and older, an increase in falls of 0.7% (95% CI: from 0.4 to -1.1) would be expected due to the age increase of one year (see supplementary figure.s1), i.e., 6 additional falls (or 130 falls in total) in the following year would have taken place without a fall prevention program.

In fact, in the year after the home visit, 55 participants had a total of 67 severe falls. Compared to the expected 130 severe falls, the actual number of falls decreased by 63 (-48%). This decrease was concentrated on participants experiencing only one severe fall per year. Based on accident insurance claims related to falls occurring before and after home visits, the average cost per fall amounted to approximately $4,600 (Table 3), the majority of which are inpatient care costs (acute care and rehabilitation). Nearly 80% of costs are generated by 40% of the falls requiring inpatient care (see supplementary figure.s2). Costs per fall were slightly higher in the year after the home visit than in the year before, but not statistically different. Thus, no evidence was found that the FPP reduced the severity of fall consequences. With program costs of $370,500 and healthcare cost savings of $288,855, the ICER (Incremental Cost-Effectiveness Ratio) was at $1,296 per severe fall prevented (see Table 3).

**Table 3:** Results of cost-effectiveness estimation

Category	Incremental
Costs (CHF)	
Program costs	370,500
Medical treatment costs (due to falls)	-288,855
	[-183,400; -366,800]
Total costs	81,645
	[187,100; 3,700]
Benefit	
Number of prevented falls	63
	[40; 80]
Cost-Benefit	
ICER = Cost per prevented fall	1,296

Notes: *N* = 741. Time horizon is one year before and after the intervention. CHF: Swiss Franc (1 CHF ≈ 1 USD). Average cost per fall CHF 4,585 (SD = 7,470). 95%-CI in square brackets. Per person program costs = CHF 500. Per person savings through prevented falls = CHF 390

**Fig. 3:**
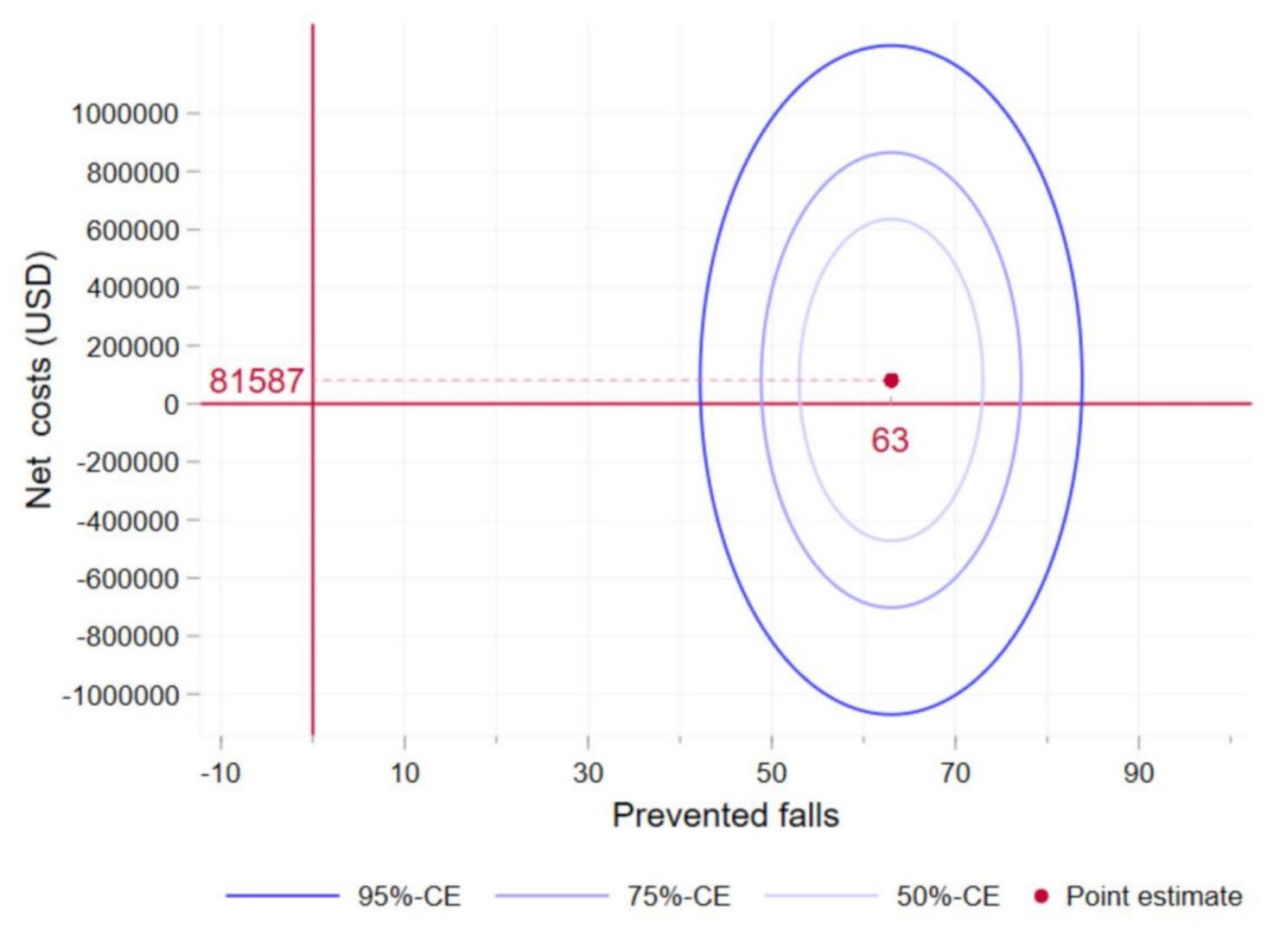
Cost-effectiveness plane and ICER ICER = incremental cost effectiveness ratio; CE = confidence ellipses Notes: This figure shows the cost-effectiveness plane with the net costs on the y-axis and the number of prevented falls on the x-axis. The ICER amounts to USD 1’296 (= 81’645 / 63). The confidence ellipses are drawn at the 50%, 75% and 95%-Level

Figure 3 illustrates the confidence ellipses of the ICER in the cost-effectiveness plane. The large portion of the confidence contours below the horizontal axis indicates that the program has a nearly 50% probability of being cost saving from an accident insurance perspective.

## Discussion

This study found a substantial reduction in the fall rate of older people in the year after the home visit. The evaluation also showed small, but statistically significant, short- and long-term effects on fear of falling, quality of life, physical activity, self-efficacy, and adherence. Although these results are ‘only’ based on pre-post analysis, with well-known limitations, the decrease in the fall rate was further reflected in the insurance claims data with a reduction in the number of severe falls requiring medical care of minus 48% at a cost of $1,296 per prevented fall. Sensitivity analysis showed a probability of nearly 50% that the programme is cost saving.

The recall of falls is a challenge and probably affected by bias, as outlined in the methods. Interestingly, underreporting seems not to increase over time. It was found to not increase further from 23%, respectively 13%, at 3 or 6 months compared to 12 months [26, 27]. Cummings suggested that this might be due to the difficulty of placing fall events in a specific period of time and concluded that a longer period of time may be more likely to capture a better level of recall [27]. MacKenzie called this phenomenon ‘‘telescoping’’, where a person recalled a special event even when it hadn’t occurred during the recall period [25]. Our solution to this recall-bias problem was to weight the two self-reported counts, with the aim of achieving a conservative estimate of the fall rate reduction. Accurate assessment of falls during the observation period is also critical in determining the rate of fall reduction. Therefore, a calendar of falls, considered as the gold standard in prospective studies [24], was used in combination with the bi-monthly telephone calls.

A recent systematic review [29] confirmed the 2012 Cochrane review results on the effectiveness of multifactorial interventions in preventing falls in community dwellers, mostly aged 65 years or older, and identified studies with even larger effects. The 28 studies with multifactorial interventions significantly reduced the fall rates in the intervention groups compared to the control groups, with a risk ratio of 0.68 (95% CI from 0.58 to 0.81). Subgroup analysis based on the inclusion of environmental modifications in the intervention showed that multifactorial interventions with environmental modifications significantly reduced fall rates (risk ratio 0.65 (95% CI from 0.54 to 0.79) compared to usual care. Interestingly, multifactorial interventions without environmental modifications were not better than usual care, i.e. not effective (risk ratio 0.82 (95% CI from 0.55 to 1.21). Mean age of participants in majority of studies was around 80 years, therefore comparable with our study, however most of those interventions lasted at least several weeks, and most up to one year. It is therefore remarkable that the SDL’s low-threshold intervention with one single home visit achieved its remarkable benefits. In fact, the SDL seems to have consequently and successfully transferred previous evidence into practice.

Equally remarkable is the reduction in severe falls estimated from claims data, showing that only 12 home visits are needed to prevent one severe fall. Several systematic reviews have explored the cost-effectiveness of FPPs using cost per quality-adjusted life-year (QALY) gained [30], cost per fall prevented [31], or both [13] Our estimated ICER of 1296 USD per fall prevented is at the lower bound of the 31 economic evaluations of FPPs in the latter review. This is also well below the willingness-to-pay threshold of 5,000 Canadian dollars per fall prevented, as determined in a recent study [32]. In this context, it must be remembered that our result relates solely to falls requiring medical treatment, and not to all falls incurred. Furthermore, we believe that our result is a conservative estimate of cost effectiveness. Firstly, no consultation costs with the family doctor, physio-or occupational therapist were included. Secondly, for treatments lasting longer than 2 months, the costs incurred beyond the 2-month time threshold were not included. Finally, the substantial costs due to nursing home placements triggered by severe falls were not considered. We believe that these cost underestimations would largely compensate for any overestimation of fall costs caused by the potential inclusion of treatment costs arising from further accidents taking place during the 2 months after a fall.

The participants were 82 years old on average. At this age, due to a possible decrease in health, one year more may have a direct impact on the risk of falling. Interestingly, the perceived health related quality of life remained unchanged, whereas the positive effects on fear of falling, health-related quality of life, and physical activity were found not only in the 4 months after the intervention, but throughout the observation period, even though adherence to the recommendations and exercises decreased over time.

Given these findings, the question must be asked as to whether a FPP, such the SDL, should be recommended to all older people. The pilot SDL’s qualitative evaluation revealed that physiotherapists and general practitioners favored inviting all older people to a FPP to prevent first falls, but they were concerned that this might upset them. In fact, older persons felt no need to attend the FPP before experiencing a fall [17]. Although most studies include participants above 60 or 70 years [29, 33], mean age of participants is usually in their upper seventies or lower eighties, as in the SDL. Thus, older people may perceive the need for a FPP later than health care providers, and only when they worry themselves about their gait security, irrespective of a previous fall [34]. Recently, a predictor model with good internal and external validity, based on community dwelling older adults, was developed that might assist clinicians to identify those at high risk of falls [35, 36]. The ten predictors identified ‘history of falls in the previous year’ as the strongest predictor (OR = 2.05 (95%CI from 1.88 to2.23), other important predictors were age, being female, specific medication, and cognitive problems.

Strengths and limitations: the 1-year follow up of participants and the complementary evaluation of effectiveness and cost- effectiveness based on different data sets is a strength of this study. The conservative estimation method of weighting the means of falls at one year prior and one month prior resulted in a decreased likelihood of overestimating the fall reduction rate. A further strength is the prospective assessment of falls. These strengths may outweigh the design of this study and the missing control group that must be considered as the major limitation. In fact, participants self-referred to the SDL and the study was only an add-on. Therefore, it was not possible to randomize to ‘control’ or even waiting list, also taking into account the need given the identified fall risk and the age of the participants.

However, the participants represented a special group of older adults with non-mandatory insurance, thus with potentially higher socio-economic status; and, as self-referrers, they maybe were especially motivated. Both facts may indicate selection bias. However, motivation is important, and as discussed above, older people may perceive a need for a FBB only when they worry about their gait security.

The self-reporting of falls by the participants might also be considered a limitation, however, the combination of calendar-based fall monitoring and bi-monthly telephone contact was considered the best solution for assessing the number of falls accurately, given limited financial resources, load on staff and the age of the participants. It is unclear whether bi-weekly or monthly contact would have been better than bi-monthly because many participants complained about the number of telephone calls or did not answer calls if they didn’t recognize the number, and several telephone calls were often necessary to make contact. The staff performing the calls was very committed and collected the data for all 639-person follow-ups over one year, without missing values. In turn, social desirability may have occurred when answering the questionnaires orally, even though the staff calling the participants was not involved in the study. Thus, improvements in our study may partly be due to increased awareness of risk of falling, however, the elimination of environmental and other extrinsic fall risk factors and the long-term positive effects on fear of falling, health-related quality of life and physical activity indicate also ‘real changes’.

Finally, the medical costs covered by the accident insurance accrued in the first two months following a fall were used as an approximation of the total fall cost. This approximation might differ from the true cost of a fall because the choice of the time window entails a trade-off: a shorter duration (e.g. 1 month) results in incomplete data for longer-lasting medical cases; a longer duration (e.g. 6 months) increases the risk of a recurrent accident and the inclusion of costs unrelated to the initial fall. By setting the time window at two months, the result is considered a conservative estimate.

## Conclusions

The SDL can potentially have a large impact on both individual care and public health. The following specific strategies for the systematic implementation of low-threshold models, such as the SDL, are suggested: (1) Use of a prediction model in routine care to improve the identification of community-dwelling older people at high risk of falls; (2) Targeting individual and systemic barriers and facilitators to increase the acceptance of FPPs as a measure to preserve safe mobility and autonomy; and, most importantly: (3) Secure financing of FPPs. Most health systems do not currently finance preventive interventions. However, there is an urgent need for innovative, effective and cost-effective models of care, given demographic development and the magnitude of the individual and societal burden from falls.

## Electronic supplementary material

Below is the link to the electronic supplementary material.

Supplementary Material 1

Supplementary Material 2

## Data Availability

Raw data of the health data are owned by the patient organisation, the Swiss League Against Rheumatism SLAR, and are not publicly available to preserve individuals’ privacy under the European General Data Protection Regulation. However the data analysis protocol can be requested from the first author. Raw data for the cost data are owned by the particpating health insurer companies, and are not publicly available to preserve individuals’ privacy under the European General Data Protection Regulation.

## Declarations

### Previous presentations

The study was presented orally at the (virtual) EULAR conference 2021 (health professionals’ abstract session, OP0158-HPR-EULAR-2021).

A summary of the project was provided within the project report to the SLAR and AGE foundation (*Sturzstudie.pdf (rheumaliga.ch)*.

## Abbreviations

CHF: Swiss Francs
CI: Confidence Interval
EQ-5D-5 L: EuroQuol-5 Dimensions-5 Levels
FCST: Five Chair Stand Test
FES-I: Falls Efficacy Scale-International
FPP: Fall Prevention Programme
GEE: Generalized Estimating Equations
ICER: Incremental Cost-Effectiveness Ratio
IPAQ: International Physical Activity Questionnaire
MHI: Mandatory health insurance
PA: Physical Activity
PGICS: Patients’ Global Impression of Change Scale
SDL: Safe through Daily Life
SHS: Swiss Health Survey
SLAR: The Swiss League Against Rheumatism
TUG: Timed Up and Go
US: United States
USD: United States Dollar
