# Supplementary material for: Effectiveness and cost-effectiveness of a single home-based fall prevention program: a prospective observational study based on questionnaires and claims data
Source: BMC Geriatr. 2024 Dec 28;24:1044. doi: 10.1186/s12877-024-05586-x (PMC11681629; doi:10.1186/s12877-024-05586-x)
Supplement: Supplementary file 1 — Supplementary Material 1 [file 12877_2024_5586_MOESM1_ESM.docx]

**Additional file**

**Figure.s1:** Estimated increase in risk of falling (age 65 year and over)

The dots show the share of persons with at least one fall per year by age (65+ years). The solid line shows the results of the linear regression where the share of persons with at least one fall per year is regressed on age. Its slope (coef. = 0.714) represents the estimated average increase (in percentage points) in the risk of falling per year due to an increase in age by one year.
