# Supplementary figures and images for: Effectiveness and cost-effectiveness of a single home-based fall prevention program: a prospective observational study based on questionnaires and claims data
Source: BMC Geriatr. 2024 Dec 28;24:1044. doi: 10.1186/s12877-024-05586-x (PMC11681629; doi:10.1186/s12877-024-05586-x)

**Additional file**

**Figure.s2:** Cost composition per fall


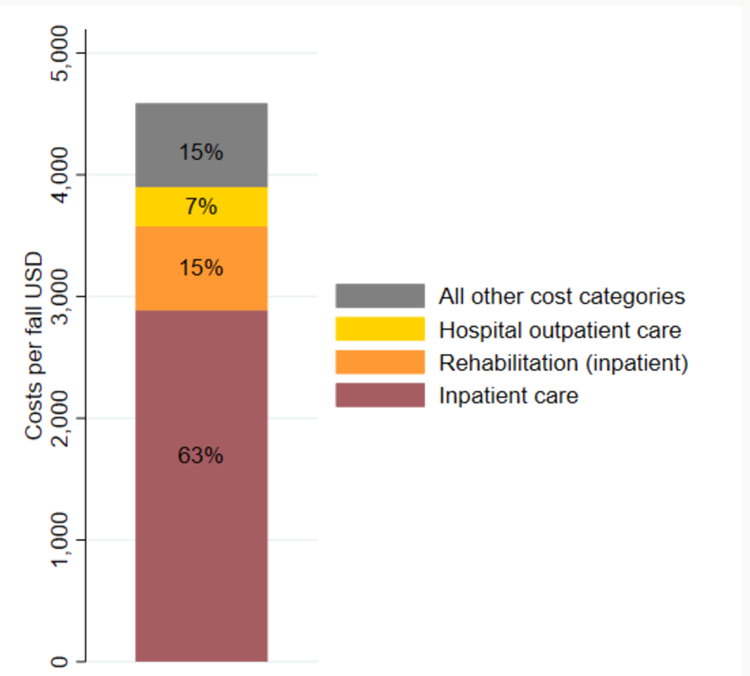

Supplement: Supplementary file 2 — Supplementary Material 2 [file 12877_2024_5586_MOESM2_ESM.docx]
